# Association of quality of life with marital satisfaction, stress, and anxiety in middle-aged women

**DOI:** 10.3389/fpsyg.2024.1357320

**Published:** 2024-09-03

**Authors:** Tayebeh Rakhshani, Masoumeh Amirsafavi, Nasrin Motazedian, Pooyan Afzali Harsini, Amirhossein Kamyab, Ali Khani Jeihooni

**Affiliations:** ^1^Department of Public Health, School of Health, Nutrition Research Center, Shiraz University of Medical Sciences, Shiraz, Iran; ^2^Department of Public Health, School of Health, Shiraz University of Medical Sciences, Shiraz, Iran; ^3^Shiraz Transplant Research Center, Shiraz University of Medical Sciences, Shiraz, Iran; ^4^Department of Public Health, School of Health, Kermanshah University of Medical Sciences, Kermanshah, Iran; ^5^Faculty of Medicine, Fasa University of Medical Sciences, Fasa, Iran

**Keywords:** quality of life, marital satisfaction, middle-aged women, stress, anxiety

## Abstract

**Background:**

Marital satisfaction is one of the important components of quality of life. Women’s marital satisfaction is affected when they enter the middle age period, due to the mental and emotional tensions caused by the physical changes. In this regard, the present study aimed to investigate the association of quality of life with marital satisfaction, stress, and anxiety in middle-aged women referring to health centers of Ahvaz city, Iran.

**Methods:**

This cross-sectional descriptive-analytical study was conducted on 1,000 middle-aged married women (30–59 year of age) under the auspices of health centers of Ahvaz city, Iran in 2019. The subjects were selected by simple random sampling method, and were asked to complete demographic characteristics, quality of life questionnaire, Enrich marital satisfaction questionnaire, Holmes-Raheh stress questionnaire, and Spielberger state–trait anxiety inventory. The data were analyzed by using SPSS 0.22 software through mean, standard deviation, frequency, Pearson correlation and regression (*p* = 0.05).

**Results:**

Based on the results, 42.4% of the participants were between 40 and 50 years of age, 35.6% had a high school diploma, and 50% of them were housewives. Also, the results of Pearson’s correlation showed a positive and significant relationship between quality of life and marital satisfaction (*r* = 0.178) (*p* < 0.001). However, quality of life had a negative and significant relationship with anxiety (*r* = −0.552) (*r* < 0.001) and stress (*r* = −0.188) (*p* < 0.001).

**Conclusion:**

Given the positive and significant relationship between quality of life and marital satisfaction, appropriate trainings are highly recommended for couples to increase the quality of life and marital satisfaction of middle-aged women and thus strengthen the health of family and society.

## Background

As a social, emotional unit, the family is one of the most important institutions of society and personality-shaper (Mannino and Schiera, 2017; Moghadasali et al., 2021). Among the factors affecting the strength of family is the quality of life (Heidary, 2018). According to World Health Organization, quality of life is a person’s understanding of his/her position in life, in the cultural context and value systems in which he/she lives, and defined his/her standards and interests in relation to goals and expectations, which includes physical, psychological dimensions, level of independence, social relations, connection with the environment, and spirituality (Mannino et al., 2019a). On the other hand, the quality of marital life is considered as one of the subsets of quality of life (Rycroft, 2018). The quality of life in marital life is a process that is determined by the degree of marital conflict, satisfaction and agreement in decision-making (Karami et al., 2021). Marital satisfaction not only affects the physical and mental health of couple, but it also influences the growth of children, good biological functions, academic performance, social skills and relationships (Bandelow and Michaelis, 2015). One of the consequences of the decrease in marital satisfaction is the increase in the number of divorces (Rahimi et al., 2018); for example, according to the report of the Civil Registration Organization in the first 6 months of 2014, the ratio of marriages to divorces was 1:4.4 (Asgari and Torkashvand, 2018).

Several studies have been conducted on marital satisfaction and its affecting factors (Mannino et al., 2019b). A study by Hamilton measured marital satisfaction in different periods of life up to old age and showed that marital relationships change throughout the life. After Hamilton, various studies showed that the relationship between life cycle and marital satisfaction follows a U-shaped curve, characterized by high marital satisfaction in the first years of marriage before becoming a parent, a decrease in marital satisfaction in the middle years, and then an increase in marital satisfaction in the final years of life (Zarei et al., 2019). In this regard, Robles et al. acknowledged that marital conflicts directly increase depression symptoms and dysfunction and thus depression. For this reason, marital conflict is an important and dangerous factor for mental health in middle age and old age (Robles et al., 2014). In a study by Karami et al. (2021), the quality-of-life increased marital life skills and, as a result, marital satisfaction.

In the family system, women are more vulnerable than other members (Asgari and Torkashvand, 2018). They are more susceptible to mental disorders due to their different roles as wives, mothers and family caregivers. Anxiety disorders are therefore the most common class of disorders in women (Sahami and Amini-Manesh, 2019). On the other hand, with the passage of time and the entry of women into the middle-age period, they may be exposed to different physical changes (Kamalian et al., 2021) leading to a decrease in self-confidence, life skills, feeling of usefulness, quality of life, and increased level of depression and mental problems (Thompson and Bardone-Cone, 2019). In addition, stress and anxiety are the most important psycho-social factors associated with this period of life (Nigdelis et al., 2018), emphasizing the importance of middle age in women’s life (Karvonen-Gutierrez and Kim, 2016).

Considering these issues, association of mental health with marital satisfaction (Rahimi et al., 2018), and destructive effect of an unsuccessful marriage on the mental health of family, investigation of marital satisfaction and its various dimensions is highly recommended (Asgari and Torkashvand, 2018). Given the complex nature of quality of life and marital satisfaction and their importance in health studies, the present study aimed to investigate the association of quality of life with marital satisfaction, stress, and anxiety in middle-aged women referring to health centers of Ahvaz.

## Methods

This cross-sectional descriptive-analytical study. The population studied in this research were middle-aged married women who referred to health centers in Ahvaz city, Iran in 2019. First, the sample size of the research is based on similar studies (Javdan et al., 2018) and the confidence level is 90% for a survey (cross-sectional) study with the formula 1,000 people were selected.


$$n=\frac{{Nz}^{\gamma }pq}{{Nd}^{\gamma }+z^{\gamma }pq}$$

This cross-sectional descriptive-analytical study was conducted on 1,000 middle-aged married women (30–59 year of age) under the auspices of health centers of Ahvaz city in 2019. According to the location of the health centers, Ahvaz was divided into two regions: east and west. Then, out of 19 health centers in the eastern region, 9 centers and out of 15 health centers in the western region, 7 centers were randomly selected.

## Inclusion and exclusion criteria

Inclusion criteria for the study were as follows: married women aged 30–59 years, no sexually transmitted diseases, no diagnosed mental health problems, no chronic diseases such as diabetes and hypothyroidism, no addictions, no history of cosmetic surgery, minimum literacy level, and not requesting a divorce. Participants were required to complete a written informed consent form to participate in the study.

Exclusion criteria were designed to ensure the reliability and accuracy of the data collected. Women who failed to answer all the questions in the questionnaires were excluded. Additionally, women with any ongoing severe medical conditions not specified in the inclusion criteria that could significantly impact their quality of life or marital satisfaction were excluded. Women currently undergoing any form of psychiatric treatment or therapy were also excluded to eliminate the potential influence of these treatments on their reported stress, anxiety, and quality of life. Furthermore, women with recent significant life events (e.g., death of a close family member, divorce proceedings) that could cause acute stress were excluded to maintain a stable baseline for measuring stress and anxiety. Lastly, women who were unwilling or unable to provide consent were excluded from the study.

## Data collection tool

Data collection tool included demographic characteristic, quality of life questionnaire, Enrich marital satisfaction questionnaire, Holmes-Raheh stress questionnaire, and Spielberger state–trait anxiety inventory.

### Demographic characteristics

Demographic characteristic included age, marital status, education, employment, age of marriage, duration of marriage, and number of children.

### Quality of life questionnaire

The Quality-of-Life Questionnaire was compiled by the World Health Organization to check people’s quality of life. This questionnaire contains 26 five-choice questions (very dissatisfied to very satisfied). The lowest score that any person can get is 26 and the highest score is 130. A higher score indicates a higher quality of life (Karami et al., 2021). In a study by Lavy and Litman-Ovadia, the reliability of the questionnaire was calculated using three retest methods with an interval of 3 weeks, dividing by two halves, and Cronbach’s alpha coefficient was 0.67, 0.87, and 0.88, respectively. The validity of the tool was investigated using differential and structural method, and was reported as acceptable (Lavy and Littman-Ovadia, 2011). In Iran, this questionnaire was translated and standardized by Nejat et al. (2006). The reliability of the questionnaire was obtained above 0.7. In a study by Karami et al. (2021), the overall reliability of the questionnaire was obtained using Cronbach’s alpha method as 0.78.

### Enrich marital satisfaction questionnaire

This questionnaire has 47 questions and consists of 12 scales that include contractual answers, marital satisfaction, personality issues, marital relationship, conflict resolution, financial management, leisure activities, sexual relations, marriage and children, relatives and friends, roles related to equality between men and women, and ideological bias (Karami et al., 2021). In this study, a short questionnaire containing 35 questions was used. The scales of the questionnaire included ideal distortion, marital satisfaction, communication, and conflict resolution. Favers and Olson calculated the total Cronbach’s alpha for this questionnaire once as 0.71 and again as 0.74. In a study by Heydari, the alpha coefficient for the whole questionnaire, marital satisfaction, communication, conflict resolution, and ideal distortion were 0.74, 0.86, 0.80, 0.84 and 0.83, respectively (Heidary, 2018). The questionnaire I based on a five-point Likert scale (completely agree, agree, neither agree nor disagree, disagree, completely disagree), with a scoring range of 35–175.

### Holmes-Raahe stress questionnaire

Consisting of 43 questions, this questionnaire as designed to show the impact of stress caused by major life changes. To determine the reliability of this scale, split-half method was used. The reliability of the questionnaire using Cronbach’s alpha was found to be 0.79 and its validity was obtained using Pearson’s correlation coefficient as 0.36 (Holmes and Rahe, 1967; Cohen, 2000). In a study by Heydari and Namjoosangari (2011), the reliability of the questionnaire was calculated using Cronbach’s alpha method and the split-half method as 0.72 and 0.64, respectively, indicating its acceptable reliability.

### Spielberger state–trait anxiety inventory

This questionnaire contains 20 questions on overt anxiety and 20 questions on covert anxiety based on a four-point Likert scale. Regarding overt anxiety, the subjects express their feelings at the same moment, and regarding covert anxiety, they express their usual feelings most of the time (Wongkietkachorn et al., 2018). In a study by Heshmatifar et al. (2020), the reliability of the questionnaire was calculated as 0.90 by using Cronbach’s alpha method.

## Ethical considerations

The present study was approved by the Ethics Committee of Shiraz University of Medical Sciences (Ethics code: IR.SUMS.REC.1399.962). A written consent was obtained from the participants and they were assured that their information would be treated strictly confidential.

## Data analysis

The data were analyzed using SPSS 22 software, employing descriptive statistics to summarize the demographic characteristics of the participants and inferential statistics to explore the relationships between variables. Descriptive statistics included means, standard deviations, and frequencies. Pearson correlation tests were used to assess the relationships between quality of life, marital satisfaction, stress, and anxiety. Interpretation of the Pearson correlation coefficients followed the guidelines: values close to 1 or − 1 indicated a strong positive or negative relationship, respectively, while values around 0 indicated no relationship.

For more in-depth analysis, multiple linear regression (MLR) was employed to examine the predictive power of stress, anxiety, and marital satisfaction on quality of life. Assumptions for MLR were rigorously tested to ensure the validity of the model. Multicollinearity was assessed using the Variance Inflation Factor (VIF), with a VIF value exceeding 10 indicating significant multicollinearity among the predictors. The Durbin-Watson test was used to check for autocorrelation in the residuals, with values close to 2 suggesting no autocorrelation. Additionally, Cook’s distance was monitored to identify potential influential data points, with values exceeding 1 indicating significant influence on the regression model. These diagnostics ensured the robustness and reliability of the regression analyses (*p* = 0.05).

## Results

Based on the results, 42.4% of the participants were 40–50 years of age. Also, most of them had a high school diploma (35.6%) and were housewives (50%). 44.3% of the participants were married between the ages of 15 and 20, and 52% were married for 25–30 years. 33.1%of the children had more than 5 children. 34.7% of the participants’ wives had middle school degree and 55.4% of the participants’ wives was worker (Table 1).

**Table 1:** Demographic characteristics of the participants.

Variable		Number	Percentage
Age	30–40	304	30.4
	40–50	424	42.4
	50–59	272	27.2
	Total	1,000	100
Education	Illiterate	108	10.8
	Secondary school	174	17.4
	High school	356	35.6
	Post-diploma	208	20.8
	M.A.	131	13.1
	B.A.	23	2.3
	Total	1,000	100
Employment	Housewife	500	50
	Employed	100	10
	Self-employed	98	9.8
	Retired	41	4.1
	Worker	160	16.1
	Other	101	10.1
	Total	1,000	100
Age of marriage	10–15	157	15.7
	15–20	443	44.3
	20–25	101	10.1
	25–30	150	15
	30–35	79	7.9
	35–40	70	7
	Total	1,000	100
Duration of marriage	10	149	14.9
	15–20	331	33.1
	25–30	520	52
	Total	1,000	100
Number of children	1–3	266	26.6
	3–5	403	40.3
	≥5	331	33.1
	Total	1,000	100
Wife’s education	Illiterate	56	5.6
	Secondary school	347	34.7
	High school	205	20.5
	Post-diploma	201	20.1
	M.A.	185	18.5
	B.A.	6	6
	Total	1,000	100
Wife’s employment	Employed	199	19.9
	Self-employed	123	12.3
	Retired	124	12.4
	Worker	554	55.4

The mean score of quality of life, marital satisfaction, stress and anxiety was 79.17 ± 87.35, 139.29 ± 21.77, 109.25 ± 18.28, and 113.25 ± 25.52, respectively.

Based on the Table 2, quality of life had a positive and significant relationship with age (*r* = 0.421) (*p* < 0.001), education (*r* = 0.385) (*p* < 0.001), employment (*r* = 0.568) (*p* < 0.001), time of marriage (*r* = 0.520) (*p* < 0.001), duration of marriage (*r* = 0.193) (*p* < 0.001), number of children (*r* = 0.397) (*p* < 0.001), wife’s education (*r* = 0.374) (*p* < 0.001), and wife’s employment, (*r* = 0.230) (*p* < 0.001). Also, marital satisfaction had a positive and significant relationship with age (r = 0.262) (p < 0.001), education (*r* = 0.290) (*p* < 0.001), employment (*r* = 0.171), (*p* < 0.001), time of marriage (*r* = 0.222) (*p* < 0.001), duration of marriage (*r* = 0.327) (*p* < 0.001), number of children (*r* = 0.285) (*p* < 0.001), wife’s education (*r* = 0.233) (*p* < 0.001), and wife’s employment (*r* = 0.325) (*p* < 0.001).

**Table 2:** The results of Pearson correlation of quality life, marital satisfaction, and demographic characteristics.

Variable	Marital satisfaction	Quality of life
Age	*r* = 0.262	*r* = 0.412
	*p* ≤ 0.001	*p* ≤ 0.001
Education	*r* = 0.290	*r* = 0.385
	*p* ≤ 0.001	*p* ≤ 0.001
Employment	*r* = 0.171	*r* = 0.568
	*p* ≤ 0.001	*p* ≤ 0.001
Age of marriage	*r* = 0.222	*r* = 0.520
	*p* ≤ 0.001	*p* ≤ 0.001
Duration of marriage	*r* = 0.327	*r* = 0.193
	*p* ≤ 0.001	*p* ≤ 0.001
Number of children	*r* = 0.285	*r* = 0.397
	*p* ≤ 0.001	*p* ≤ 0.001
Wife’s education	*r* = 0.233	*r* = 0.374
	*p* ≤ 0.001	*p* ≤ 0.001
Wife’s employment	*r* = 0.325	*r* = 0.230
	*p* ≤ 0.001	*p* ≤ 0.001

Based on the Table 3, quality of life had a positive and significant relationship with marital satisfaction (*r* = 0.178) (*p* < 0.001). However, quality of life had a negative and significant relationship with anxiety (*r* = −0.552) (*p* < 0.001) and stress (*r* = −0.188) (*p* < 0.001).

**Table 3:** The results of Pearson correlation of quality life, marital satisfaction, stress and anxiety.

Variable	Quality of life
Marital satisfaction	*r* = 0.178
	*p* ≤ 0.001
Stress	*r* = −0.188
	*p* ≤ 0.001
Anxiety	*r* = 0 = −0.552
	*p* ≤ 0.001

Based on the Table 4, the correlation between the independent variables and the dependent variable was 0.601. According to the value obtained for the coefficient of determination, it can be said that approximately 36% of the quality of life was related to the marital satisfaction. Since this value does not consider the degree of freedom, therefore, the adjusted coefficient of determination was used for this purpose, which was equal to 36% in this test. The standard error of the estimate indicates the predictive power of the regression equation. The mentioned model shows that the variable of marital satisfaction could significantly determine 36% of the quality of life. Also, stress and anxiety variables could significantly determine 33 and 30% of the quality of life, respectively (*p* < 0.05).

**Table 4:** Regression coefficients of marital satisfaction, stress, and anxiety.

Predictive variable	Multiple correlation	Coefficient of determination	F ratio	Regression coefficients
Anxiety	0.552	0.305	437.22	B = 0.36 T = 21.15 *p* = 0.001
Stress	0.557	0.333	249.37	B = 0.175 T = 7.25 *p* = 0.001
Marital satisfaction	0.601	0.361	187.36	B = 0.132 T = 6.52 *p* = 0.001

## Discussion

The primary objective of this study was to examine the correlation between quality of life and marital satisfaction, tension, and anxiety in middle-aged women who seek assistance from health clinics in Ahvaz. The findings indicated a positive and substantial correlation between quality of life and demographic factors including age, education, employment, age of marriage, duration of marriage, number of children, wife’s education, and wife’s work. These findings align with the results of a study conducted by Kim and Kang (2015). Research has indicated that the overall well-being of individuals typically reaches its highest point during the initial years of marriage, but gradually decreases until middle age. However, from that point onwards, it gradually improves with advancing age and the length of the marital union (Shahbazi and Khademali, 2018). Furthermore, both education and employment have the potential to significantly impact the overall well-being and health-consciousness of the family. Prior research has demonstrated a positive correlation between income level and quality of life (Amirzadeh-Iranagh et al., 2021).

The findings of this study indicate a clear and meaningful correlation between marital satisfaction and demographic factors including age, education, employment, age at marriage, length of marriage, number of children, wife’s education, and wife’s work. These findings align with the results of the study conducted by Robles et al. (2014). Nevertheless, the findings of a study conducted by Rajaei Ghazilo et al. (2021) indicate that working women experience greater drawbacks in terms of life happiness and marital satisfaction compared to those who focus on housekeeping. According to a study conducted by Shahbazi and Khademali (2018), women who were married for less than or equal to 5 years reported higher levels of marital satisfaction compared to women who were married for 5 years or more. In a study conducted by Dabone (2014), it was found that there was no statistically significant disparity in marital satisfaction between young and senior couples. According to a study conducted by Chao et al. (2011), there is a decline in women’s sexual desire and sexual satisfaction as they age. Additionally, the study found that elderly women have lower levels of sexual desire and satisfaction compared to middle-aged women. The outcomes of the present study were incongruous with these results. However, researchers have discovered that marital satisfaction typically follows a U-shaped pattern. This pattern is characterized by a high level of satisfaction in the initial years of marriage, followed by a decline in satisfaction during the middle years, and finally an increase in satisfaction during the later years of life (Rajabi et al., 2021). This is due to the extended life expectancy and aging of spouses, which leads to the resolution of many behavioral differences and obstacles faced by couples, resulting in an improvement in their psychological well-being.

The present study found a strong and statistically significant correlation between the quality of life and marital satisfaction. This finding is consistent with previous studies conducted by Heidary (2018), Sohrabi et al. (2016), Karami et al. (2021), Tavasoli and Nava (2017), Kim and Kang (2015), Robles et al. (2014), and Chao et al. (2011). Women in their middle age may experience heightened levels of strain and pressure. Stress and anxiety are prevalent problems in contemporary times (Emdadi et al., 2018). Stress and anxiety significantly impact the overall quality of life. Furthermore, the impact of psychological factors on the physical dimensions of quality of life has been demonstrated (Stefanaki et al., 2015). The current study found a strong and negative correlation between quality and anxiety and stress. This suggests that managing and addressing women’s stress and anxiety can have a significant impact on their mental well-being, quality of life, and marital satisfaction (Ekici et al., 2017). The findings were in line with the results of research conducted by Sohrabi et al. (2016). The study conducted by Bahmani-makavandzadeh and Amanelahi (2018) found that marital instability, marital satisfaction, and marital conflicts are indicators of depression in married women. The study conducted by Kim and Kang (2015) revealed a substantial negative association between depression and quality of life. Furthermore, stressful circumstances played a significant role in predicting and impacting the quality of life of individuals in their middle age. According to Robles et al. (2014), marital disagreements have a significant and hazardous impact on mental health during middle age and old age.

Given that women play a crucial role in societal development and family well-being, they have significant responsibilities within the family. However, in order to perform these tasks, it is essential for women to maintain optimal physical and mental health (Mannino and Caronia, 2017). Unfortunately, mental diseases place a substantial burden on women. Therefore, implementing self-confidence enhancement sessions, stress management programs, and awareness campaigns can be efficacious in mitigating anxiety and stress in women. In addition, it is recommended that women be offered methods to alleviate worry and stress, as well as education on marital relationships, alongside family planning initiatives (Ghani-far et al., 2015). Insufficient proficiency in marital relations has been discovered to result in a decline in quality of life and ultimately the dissolution of the couple’s relationship (Rosenbaum and White, 2015). Hence, considering the significance of this matter, it is recommended to implement educational initiatives that focus on exploring proficient spousal communication, fostering heightened intimacy between partners, and enhancing the overall quality of marital relationships. Being in a fulfilling romantic relationship is a powerful indicator of overall life satisfaction and well-being, and it is linked to improved physical health and increased longevity (Forouzesh Yekta et al., 2018). Undoubtedly, a culture characterized by robust familial units is unequivocally a healthy society. Hence, the well-being of family members and their interpersonal connections, particularly marriage relationships, undeniably yield beneficial impacts on both the family unit and society as a whole (Shahsiah et al., 2011).

### Limitations

There are several limitations that should be acknowledged. Firstly, the study’s cross-sectional design limits the ability to infer causality between the variables. Longitudinal studies would be more effective in establishing causal relationships. Secondly, the data collected were based on self-reported questionnaires, which might introduce bias due to participants’ potential over- or under-reporting of their quality of life, marital satisfaction, stress, and anxiety levels.

Additionally, although the sample size is large, the use of simple random sampling from health centers in Ahvaz city may not fully represent the broader population of middle-aged women in Iran. The results may not be generalizable to women from different regions or socio-economic backgrounds. Furthermore, the study was conducted in a specific cultural context, which may influence the generalizability of the findings to other cultures. Cultural factors play a significant role in marital satisfaction and quality of life, and these findings may not be applicable in different cultural settings. Lastly, the exclusion criteria, such as excluding women with chronic diseases, addictions, or mental health problems, may limit the generalizability of the findings to all middle-aged women, as those with these conditions might experience different levels of marital satisfaction and quality of life.

## Conclusion

The results of the present study showed that there is a positive and significant relationship between quality of life and marital satisfaction. However, quality of life had a negative and significant relationship with stress and anxiety. Considering the very important role of women in the family, paying attention to women especially during middle age is of great importance. Marital satisfaction is one of the most influential factors in the quality of life and a healthy family, which can be improved by proper and continuous training to couples, especially women on the verge of middle age.

## Data Availability

The raw data supporting the conclusions of this article will be made available by the authors, without undue reservation.
